# Beyond batch experiments: unveiling the potential of bimetallic carbon xerogels for catalytic wet peroxide oxidation of hospital wastewater in continuous mode

**DOI:** 10.1007/s11356-024-35546-2

**Published:** 2024-11-22

**Authors:** Adriano Santos Silva, Fernanda Fontana Roman, Rui Sérgio Ribeiro, Juan Garcia, Helder Teixeira Gomes

**Affiliations:** 1 Centro de Investigação de Montanha (CIMO), Instituto Politécnico de Bragança, Campus de Santa Apolónia, 5300-253-253 Bragança, Portugal; 2 Laboratório Associado Para a Sustentabilidade E Tecnologia Em Regiões de Montanha (SusTEC), Instituto Politécnico de Bragança, Campus de Santa Apolónia, 5300 253 Bragança, Portugal; 3 LSRE-LCM – Laboratory of Separation and Reaction Engineering - Laboratory of Catalysis and Materials, Faculty of Engineering, University of Porto, Rua Dr. Roberto Frias, 4200-465 Porto, Portugal; 4 ALiCE – Associate Laboratory in Chemical Engineering, Faculty of Engineering, University of Porto, Rua Dr. Roberto Frias, 4200-465 Porto, Portugal; 5 Catalysis and Separation Procecesses Group (CyPS), Chemical Engineering and Materials Department, Complutense University of Madrid, Avda. Complutense S/N, Madrid, Spain

**Keywords:** AOPs, Fenton-like, Process optimization, Hospital wastewater, Carbon catalyst, CECs

## Abstract

Single- and bimetallic carbon xerogels were prepared by incorporating iron and iron-cobalt precursors during their synthesis, respectively, and tested in the catalytic wet peroxide oxidation (CWPO) of ibuprofen spiked into a simulated matrix in batch mode. The bimetallic catalyst outperformed single and non-metallic catalyst by 25 and 85% after 360 min of reaction, at mild temperature (30 °C). The best-performing catalyst was further used to treat hospital wastewater in a CWPO system operating in full continuous mode. Process optimization was carried out considering different catalyst loads, temperatures, and pH. The results obtained showed that the best conditions are initial pH 3, *T* = 80 °C, and a catalyst load of 35.4 mg cm^−3^. Having maintained values of chemical oxygen demand (COD) removals as high as 80% after 24 h of continuous operation, the results herein reported revealed the high potential of the bimetallic carbon xerogel for CWPO of hospital wastewater beyond conventional applications in batch mode. Despite some catalytic deactivation, the bimetallic carbon xerogel still delivered a mineralization degree as high as 55% of the initial total organic carbon (TOC) content of the hospital wastewater in the third 24-h cycle of CWPO in continuous mode of operation with successive catalyst reuse, as opposed to a 73% TOC removal in the first cycle. Therefore, our results open prospects for the implementation of CWPO for hospital wastewater treatment in continuous mode of operation.

**Supplementary information:**

The online version contains supplementary material available at 10.1007/s11356-024-35546-2.

## Introduction

The analytical techniques available for water quality monitoring have improved substantially, allowing the detection of a significant number of organic pollutants at lower concentrations (pg L^−1^ to µg L^−1^) in various aquatic compartments such as groundwater, surface water, and water reservoirs (Liu et al. 2020; Chakraborty et al. 2021; Rathi et al. 2021; Pot et al. 2022). These compounds come from several sources, namely, industrial wastewater, municipal wastewater, hospital wastewater, agriculture, and animal husbandry (Morin-Crini et al. 2022). Some of these compounds are usually known as contaminants of emerging concern (CECs) (Shah et al. 2020). These pollutants are not commonly monitored in the environment but have the potential to cause adverse effects on the ecosystem and human health (Noguera-Oviedo and Aga 2016). In some cases, their actual effect on the environment and health is not completely established (Sousa et al. 2018). Consequently, public concern on potential environmental and human health impacts of water pollution has also been increasing (Felis et al. 2020; Atugoda et al. 2021).

Hospital wastewaters are especially problematic, as they frequently carry significative concentrations of several CECs (Verlicchi 2021), up to 150 times higher than those typically found in municipal wastewater (Lofrano et al. 2021), with concentrations as high as 15 mg L^−1^ being reported (Vieira et al. 2021). Ibuprofen (IBU) is an example of a commonly found contaminants in hospital wastewaters (Vieira et al. 2021), also occurring in concerning concentrations in the environment worldwide (Zapata and Peñuela 1951; Gallego-Ríos and Peñuela 2021; Zhang et al. 2021). IBU is consumed in large amounts (*ca*. 15,000 Mg per year worldwide), being currently ranked among the 50 drugs that carry the highest environmental threats (Nawaz et al. 2020). Even at low concentrations, IBU presence in water severely impacts aquatic life reproduction (Gong et al. 2021; Mello et al. 2022).

Many CECs are refractory to conventional wastewater treatments such as coagulation, filtration, sedimentation, and biological processes (Starling et al. 2019; Nawaz et al. 2020). In this sense, special attention has been given to catalytic wet peroxide oxidation (CWPO) — also known as heterogeneous Fenton, as it is one technology operating in mild conditions and that can reach high organic pollutants degradation rates (Ribeiro et al. 2016b). The CWPO technology is based on the interaction of the oxidant source, hydrogen peroxide, with a suitable catalyst to promote its decomposition into reactive oxygen species (ROS) (Márquez et al. 2018). Among ROS, hydroxyl radicals (HO^•^), superoxide (O_2_•^–^), and singlet oxygen (^1^O_2_) are examples of radicals with activity in organic pollutant degradation by AOPs (Rayaroth et al. 2023). For CWPO, HO^·^ are the main responsible for pollutant degradation (Inchaurrondo et al. 2012). The technology has been extensively explored to degrade organic pollutants, and its performance has been validated considering different matrices (Huaccallo-Aguilar et al. 2021a; Silva et al. 2023, 2024), including hospital wastewater (del Álamo et al. 2020; Silva et al. 2023). In the study published by Silva et al., 100% paracetamol degradation was achieved in a spiked solution using hospital wastewater as matrix in batch mode (Silva et al. 2023). Other studies reported hospital wastewater treatment technologies in batch mode, but the evaluation of CWPO and other advanced technologies for the continuous treatment of hospital wastewaters remains largely unexplored (del Álamo et al. 2020; Li et al. 2022).

Typical CWPO catalysts are based on transition metals, mainly iron. Nevertheless, leaching may be an issue in pure metal-phases. Over the years, some works have even reported that carbon materials possess, by themselves, catalytic activity for hydrogen peroxide decomposition/reduction via hydroxyl radical formation mostly due to their electron-donor ability (Liu et al. 2018; Santos Silva et al. 2024). Some works reported the synthesis of carbon-encapsulated metal nanoparticles as a solution to decrease iron leaching (Silva et al. 2023). However, the synthesis procedures often lead to low yields (in the milligram range) and are not scalable to prepare large amounts of catalysts. An easier alternative is to support a metal phase on carbon xerogels (CX). In fact, our group already optimized a synthesis methodology leading to metal-containing CXs with high effectiveness for the CWPO of organic pollutants in simulated water matrices, when operating in batch mode (Ribeiro et al. 2016a, 2017b). However, our previous studies were mostly focused on understanding the role of the different synthesis precursors and used pre-established reaction parameters for the degradation of organic pollutants. The optimization of pH and temperature in batch mode has been already reported (Zazo et al. 2006; Bautista et al. 2011; Diaz de Tuesta et al. 2020), with pH in the range of 2–4 and temperature of 25–80 °C. Typical catalyst concentration in batch systems often range from 1 to 2.5 g L^−1^ (Santos Silva et al. 2019; Huaccallo-Aguilar et al. 2021a; Guari et al. 2022). However, the impact of these parameters in a continuous operation system using carbon xerogels as catalysts remains to be explored.

Therefore, the main goal of the current study is to evaluate the catalytic activity of the CXs previously developed by our group (Ribeiro et al. 2016a, 2017b) when employed in a CWPO system operating in continuous mode. For that purpose, batch experiments with IBU spiked into distilled water were first carried out to select the most promising catalyst among those considered, namely, bare CX, iron-doped CX (Fe/CX), and iron-cobalt-doped CX (CoFe/CX). The best-performing catalyst (i.e., the bimetallic CoFe/CX) was then used in CWPO experiments (in a packed bed) designed for the treatment of hospital wastewater in continuous mode of operation. The catalyst load in the packed bed, pH of the effluent, and reaction temperature were the operating parameters evaluated. To the best of our knowledge, only a few reports have been made on CWPO of hospital wastewaters in continuous mode of operation (del Álamo et al. 2020; Huaccallo-Aguilar et al. 2021b, c), thus highlighting the relevance of our study.

## Methodology

### Reagents

Resorcinol (99 wt.%) and cobalt(II) chloride hexahydrate (99 wt.%) were obtained from Fisher Chemical. Formaldehyde solution (37 wt.% in water, stabilized with 15 wt.% methanol) and iron(III) chloride hexahydrate (97 wt.%) were purchased from Panreac. Hydrogen peroxide (30% w/v, Fischer Chemical), titanium (IV) oxysulfate (99.99% w/v, Sigma Aldrich), sulfuric acid (98% v/v, Labkem), sodium sulfite (98 wt.%, Panreac), acetonitrile (99.9% v/v, Fisher Scientific), and ibuprofen (IBU, 99%, Tokyo Chemical Industry) were used in CWPO runs and analytical techniques.

### Carbon xerogels

The CXs employed in this study were prepared as described in our previous publication (Ribeiro et al. 2016a). Please refer to Text S1 in Supplementary Information for additional details on synthesis and characterization performed.

### CWPO experiments in batch mode

The liquid-phase oxidation reactions were carried out in a 250-mL two-necked round-bottom flask equipped with a reflux condenser. The pH of the pollutant solution ([IBU]_0_ = 30 mg L^−1^) was adjusted to 3.0 before each experiment, by means of H_2_SO_4_ (0.5 mol L^−1^) addition. Once the pH was adjusted, the flask loaded with pollutant solution was submerged into an oil bath with stirring until the desired temperature was reached (30 °C). Next, the solution remained under stirring at 30 °C, and the stoichiometric amount of H_2_O_2_ for complete mineralization of IBU was added ([H_2_O_2_]_0_ = 165 mg L^−1^). The stoichiometric amount of H_2_O_2_ for complete mineralization of IBU was calculated based on Eq. (1):

1 $${\text{C}}_{13}{\text{H}}_{18}{\text{O}}_{2}+ {33\text{H}}_{2}{\text{O}}_{2}\to {\text{13ICO}}_{2}+{42\text{H}}_{2}\text{O}$$

Prior to the addition of the catalyst, samples were withdrawn from the mixture to measure initial concentration of IBU and hydrogen peroxide. Then, 0.2 g of catalyst was loaded into the system ([cat] = 2 g L^−1^). The addition of the catalyst into the reaction medium marks the beginning of the reaction and is the reference point for the initial time (*t*_0_). The catalyst concentration, temperature, and pH were chosen based on previous studies that reported these values to be in the range for optimized H_2_O_2_ decomposition into hydroxyl radicals (Bautista et al. 2011; Diaz de Tuesta et al. 2020). The low, near room temperature used for catalyst screening (30 °C) was chosen to evidence the catalytic activity of the distinct materials. Samples were withdrawn from the reaction media in periodic times of 15, 30, 60, 120, 240, 360, 480, and 1440 min. The samples used to measure H_2_O_2_ concentration were immediately analyzed following a procedure described in our previous work (Santos Silva et al. 2019). The samples used to measure IBU concentration were stored along with *ca.* 10 mg of Na_2_SO_3_(s), which was used as a reaction inhibitor to stop the reaction. The pure adsorption experiments and non-catalytic runs were performed under the same operating conditions, but in the absence of hydrogen peroxide and catalyst, respectively.

An additional experiment was carried out to evaluate the stability of the catalyst. For that, the reaction was initiated as described above. Upon reaching 1 h of reaction, the catalyst CoFe/CX was removed from the reaction vessel by filtration. The supernatant was poured into a new reaction vessel, and the reaction was allowed to continue until the completion of the 24 h of reaction. Samples were withdrawn and analyzed as described above.

### Hospital wastewater

The hospital wastewater employed in this study was collected from a hospital located in Madrid, Spain. The effluent was characterized following the procedures reported in previous studies (Silva et al. 2023) and reported in Table 1. The pH and conductivity at room temperature were determined using WTW InoLab Cond Level 1, PHS-3BW Bench TOP pH/mV/°C meter (Bante Instruments, Shanghai, China). The total organic carbon (TOC) and total nitrogen (TN) were measured using TOC-L equipment with a nitrogen measuring unit (Shimadzu). Iron concentration was determined via atomic absorption spectroscopy (Varian SpectrAA 220) by diluting the samples with 5 wt.% HNO_3_. Chlorides were determined using the Mohr titration method. Briefly, 20 mL of the sample was titrated against 0.1 M AgNO_3_ solution using K_2_CrO_4_ as the indicator.

**Table 1:** Main characteristics of the hospital effluent used in this work

Parameters	Value
pH	8.5
TOC (mg L^−1^)	286
TN (mg L^−1^)	90.1
CO_3_^2−^ (mg L^−1^)	365.5^b^
Conductivity at 20ºC (µS cm^−1^)	2360
Aromaticity (*A*_254_ nm)	0.5
COD (mg L^−1^)	332
TDS^a^ (mg L^−1^)	0.5
Cl^−^ (mg L^−1^)	121.42
Fe (mg L^−1^)	4.6

^a^Total dissolved solids

^b^Calculated from total inorganic carbon (TIC) measurement

### CWPO experiments in continuous mode of operation

The hospital wastewater described in “Hospital wastewater” was used as matrix in all the CWPO experiments performed in continuous mode of operation using the system illustrated in Figure S1. To evaluate the optimal conditions for TOC and COD removals, the effect of catalyst load in the packed-bed (200–400 mg), temperature (30–50 °C), and initial pH (3–7.6) were studied. The values used for the parameters were based on the literature for batch CWPO experiments (Diaz de Tuesta et al. 2020). The reactor dimensions are 10 cm (length) with inner diameter of 1.2 cm. The bed height during the experiments depended on the amount of catalyst loaded, being 1 cm for 200 mg (17.7 mg cm^−3^), 1.5 cm for 300 mg (26.5 mg cm^−3^), and 2 cm for 400 mg (35.4 mg cm^−3^). The complete description of the parameters in each run is shown in Table S1. Hydrogen peroxide ([H_2_O_2_]_0_ = 706 mg L^−1^) was dosed in slight excess to that theoretically needed to meet the COD of the hospital wastewater (COD = 332 mg L^−1^). The mixture of hospital wastewater and hydrogen peroxide was fed to the reaction system at a flow rate of 1 mL min^−1^, similar to the flow used in previous continuous CWPO experiments (Silva et al. 2024). Liquid samples recovered from the reaction were analyzed by inductively coupled plasma-optical emission spectrometry (ICP-OES) to evaluate possible metal leaching.

### Analytical techniques

IBU concentration was determined by high-performance liquid chromatography (HPLC), using a Jasco HPLC system equipped with a UV/Vis detector (UV-2075 Plus), a quaternary gradient pump (PU-2089 Plus), and a Kromasil 100–5-C18 column (15 cm × 4.6 mm; 5 m particle size). The separation of the reaction products was achieved using a mobile phase composed by acetonitrile–water modified with 0.1 wt.% of H_3_PO_4_ in a volumetric ratio of 80:20. Hydrogen peroxide (H_2_O_2_) concentration was determined by a colorimetric method, as previously described (Ribeiro et al. 2015). Total organic carbon (TOC) was determined with a TOC-L CSN analyzer of Shimadzu (Kyoto, Japan). Chemical oxygen demand (COD) was determined by the colorimetric method previously reported, which takes into account the effect of residual H_2_O_2_ on COD as shown in Figure S2 (Ribeiro et al. 2017a; Freitas et al. 2022). The presence of aromatics was estimated as the absorbance obtained at 254 nm (*A*_254_).

## Results and discussion

### Characterization

The properties of the CXs used in this study were already thoroughly characterized in our previous publications (Ribeiro et al. 2016a, 2017b). The results obtained for textural properties are shown in Table S2. All the carbon xerogels have developed surface areas ranging from 510 to 650 m^2^ g^−1^. Comparing the pure carbon xerogel with the modified samples, it is possible to observe that both modifications lead to surface area decrease. For instance, modification with iron led to a 21% decrease in surface area, whereas surface area decreased by *ca.* 11% in CoFe/CX. The pristine carbon xerogel sample is mainly a mesoporous material, with a pore diameter of 6.7 nm and *V*_mic_/*V*_total_ of 0.17. The modifications have significantly influenced the pore distribution of the carbon xerogels, as can be seen with a dramatic reduction in mesoporous surface area and consequent increase in *V*_mic_/*V*_total_ values. Another consequence of the modification with metal was observed with pH_PZC_ values dropping from 9.2 in the pristine sample to 6.6 (Fe/CX) and 7.7 (CoFe/CX).

SEM analysis was conducted on carbon xerogel materials to examine their morphology. Figure S5 illustrates the morphological differences between carbon xerogels with and without embedded metal species. The material CX is depicted in Figure S5a, a standard carbon xerogel with large carbon particles (153 ± 51 mm). The large carbon xerogel observed in this figure is a result of the sol–gel polycondensation of resorcinol and formaldehyde (R/F resin). The carbon xerogel clusters are formed as a consequence of the condensation product aggregation. The main parameters controlling the carbon xerogel formation are the concentration of resorcinol and formaldehyde and the pH, as previously discussed elsewhere (Moreno-Castilla and Maldonado-Hódar 2005). On contrast, the modified carbon xerogels are composed of interconnected carbon microspheres aggregated, with metals embedded in their structure. The SEM images suggest that the embedded metals hinder the cluster aggregation, leading to the formation of smaller particles than pure carbon xerogel. The results suggest that metals’ presence in the synthetic procedure is responsible for alterations on the morphology of carbon xerogels. Other studies already reported that negatively charged functional groups of the R/F gel tend to bond with metal cations, which can act as catalysts for the polymerization reaction and influence the structure of carbon xerogels.

The XPS spectra obtained for the metal modified carbon xerogels is exhibited in Figure S6, and atomic concentrations of C 1 s, O 1 s, Fe 2p, and Co 2p are shown in Table S3. The spectra for CX/CoFe revealed that Fe 2p regions are qualitatively similar to those observed in CX/Fe. Nevertheless, the bimetallic modification in carbon xerogel led to an improved metal distribution in the surface of the resultant material, which is in agreement with previous SEM results. The simultaneous incorporation of Fe and Co results in an enhanced metal distribution on the surface of the bimetallic CX/CoFe (*cf.* Table S3). Additionally, comparing the surface weight concentrations in Table S3 to the total Fe and Co contents in Table S2 suggests that metal particles are encapsulated by the organic phase during the synthesis of monometallic CX/Fe catalyst, making them less accessible. Conversely, when Co and Fe are simultaneously incorporated into the bimetallic CX/CoFe, the metals are preferentially located on the catalyst’s surface.

### Preliminary CWPO experiments in batch mode

**Fig. 1:**
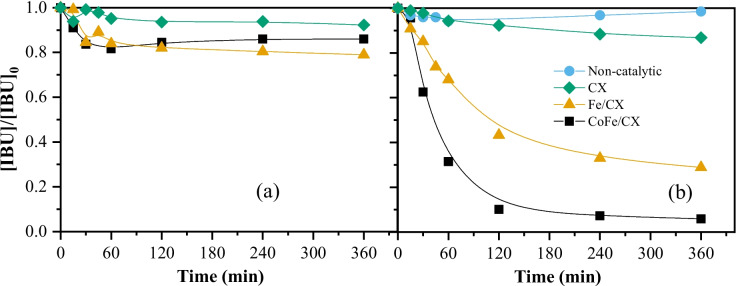
Normalized concentration of IBU during (**a**) adsorption experiments and (**b**) CWPO runs performed in batch mode. Conditions: *T* = 30 °C, pH_0_ = 3.0, [IBU]_0_ = 30 mg L^−1^, [cat] = 2 g L^−1^, [H_2_0_2_]_0_ = 165 mg L.^−1^

Pollutant removal by pure adsorption was assessed, and the results are reported in Fig. 1a. The adsorption capacity of the materials is relatively low. The *S*_BET_ seems to be slightly correlated to the adsorption capacity (*r*^2^ = 0.82, Figure S7a), whereas the pH_PZC_ seems to have a much more significant influence on the adsorption process, with an *r*^2^ of 0.98 (Figure S7b). The highest adsorption capacity (21%) was found for the slightly acidic Fe/CX (pH_PZC_ = 6.6). CoFe/CX, with a slightly basic character (pH_PZC_ = 7.7), resulted in intermediate adsorption (14%). Finally, CX resulted in the worst adsorption capacity (8%) and has a marked basic character (pH_PZC_ = 9.2).

In any case, removal of IBU by adsorption (Fig. 1a) was considerably lower compared to that obtained by CWPO (Fig. 1b), suggesting that all the materials are able to promote CWPO. In order to confirm this hypothesis, additional experiments were carried out in the absence of catalyst. The resulting non-catalytic removal curves reveal a negligible pollutant removal (Fig. 1b). The catalyst with the best performance is the bimetallic CoFe/CX (Ribeiro et al. 2017b) which enables > 90% of pollutant removal after only 120 min of reaction. Extending the reaction (for up to 6 h) increases pollutant abatement by 5%. The material composed by iron only (Fe/CX) promotes a lower IBU removal (57% and 72% after 2 and 6 h of reaction). These results agree with our previous findings on the synergistic effects arising from the simultaneous incorporation of iron and cobalt within CXs (Ribeiro et al. 2017b), allowing concluding that the better performance of CoFe/CX can be explained by the enhanced metal distribution at the surface of this bimetallic catalyst. Bare CX reveals the worst performance (Fig. 1b). This behavior is related to the higher capacity of metal-based materials to catalyze H_2_O_2_ decomposition into hydroxyl radicals (Sun et al. 2019). Still, comparing the removal of IBU obtained with CX with that obtained in the non-catalytic run confirms the catalytic features of the metal-free CX, which are mainly ascribed to the electron-donor centers (Silva et al. 2023).

Several processes for IBU removal or degradation are reported in the literature. For instance, a biological treatment with using the algae *Chlorella vulgaris* resulted in *ca.* 67% removal efficiency in 10 days of treatment (Zhou et al. 2024). Another study reported IBU adsorption up to 90% in continuous operation mode by adsorption using geopolymers (Paparo et al. 2024). Both studies are important to advance towards the removal of IBU and other pollutants from contaminated waters. However, biological treatments take considerable amount of time to present mild efficiencies, and adsorption processes are merely a transference from one phase to another.

**Fig. 2:**
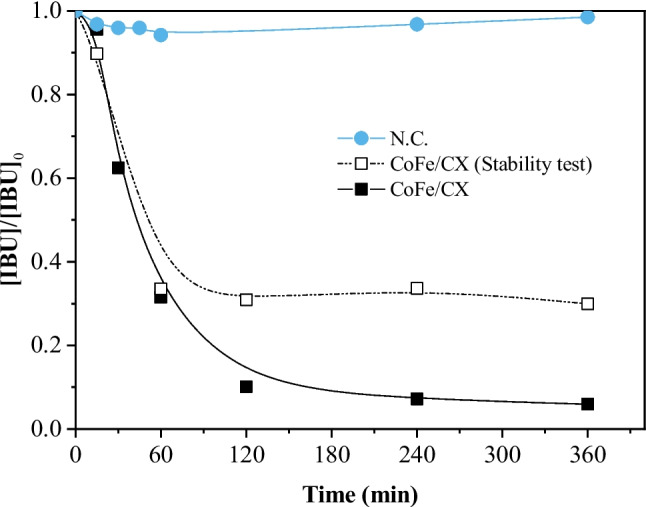
Stability test for CoFe/CX in batch mode. Conditions: *T* = 30 °C, pH_0_ = 3.0, [IBU]_0_ = 30 mg L^−1^, [CoFe/CX] = 2 g L^−1^, [H_2_0_2_]_0_ = 165 mg L.^−1^

The results of the stability assay of CoFe/CX are shown in Fig. 2. No further IBU degradation is observed after removing CoFe/CX (Fig. 2), stabilizing at 65% removal of IBU up to 6 h of reaction. For comparison, the presence of CoFe/CX until the end of the reaction resulted in a removal of IBU of 95%. This indicates that CoFe/CX is indeed a heterogeneous catalyst and not a source of homogeneous metal species that are leached into the solution and then catalyze the reaction. CoFe/CX was accordingly selected for CWPO experiments performed in continuous mode.

### CWPO of hospital wastewater in continuous mode

CWPO of the hospital wastewater described in “Hospital wastewater” was carried in full continuous mode of operation. The main properties of that wastewater are given in Table 1, confirming its representative and challenging nature. Special attention should be given to the scavenging effect of CO_3_^2−^ anions in reactions carried out with pH above 6.35, which was found to be the region where the anion has a stronger scavenging effect (Ribeiro et al. 2017a).

**Fig. 3:**
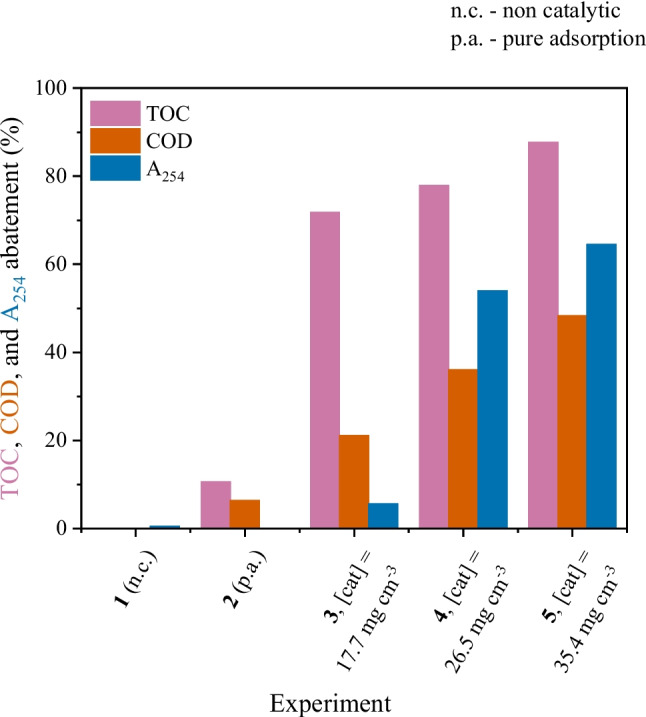
TOC, COD and A_254_ removals considering different catalyst loads after 24 h of continuous operation. Conditions: *T* = 30 °C, pH_0_ = 3.0, COD_0_ = 332 mg L^−1^, [H_2_O_2_]_0_ = 706 mg L^−1^, *m*_cat_ = 17.7–35.4 mg cm^−3^, *Q* = 1 mL min.^−1^

The results obtained for TOC, COD, and aromatics (A_254_) abatement after 24 h of continuous operation are given in Fig. 3. As with previous experiments performed in batch mode using IBU as model pollutant, negligible non-catalytic removals (of both COD, TOC, and *A*_254_) are obtained in the CWPO of hospital wastewater performed in continuous mode (Fig. 3). The adsorption run reveals that CoFe/CX can adsorb about 6.3% and 10.5% of the TOC and COD, respectively, present in the hospital wastewater. The highest COD removal (10.5%) in comparison to the TOC (6.3%) is likely related to the oxidation of other chemical species during CWPO that contribute to the oxygen demand (such as inorganic ions (Munoz et al. 2017) found in the hospital wastewater, Table 1) but do not necessarily consist of carbon. The low removals obtained in both scenarios illustrate that the system is extremely dependent on the presence of both catalyst and H_2_O_2_ to achieve higher abatement of TOC and COD. The results obtained by ICP-OES analysis revealed that no significant Fe and Co leaching were obtained in all runs, confirming the stability of the catalysts.

#### Effect of the catalyst load

Experiments were performed with different catalyst loads (17.7, 26.5, and 35.4 mg cm^−3^) inside the packed bed. As expected, increasing the amount of catalyst leads to increased removal of both TOC and COD (Fig. 3). The higher COD abatement compared to TOC removal is related to the oxidation of other chemical species rather than carbon-containing compounds, as previously reported in other studies dealing with real effluents (Freitas et al. 2022). The correlation between removal percentages and catalyst load can be described as a linear function, as seen in Figure S8a-c, with correlation coefficients (*r*^2^) of 0.98, 0.99, and 0.99 for COD, TOC, and *A*_254_ abatement, respectively. Similar results have been previously reported. Huang et al*.* have also found a positive correlation between phenol and TOC removals when increasing bed height (and, consequently, catalyst mass load) (Huang et al. 2021).

**Fig. 4:**
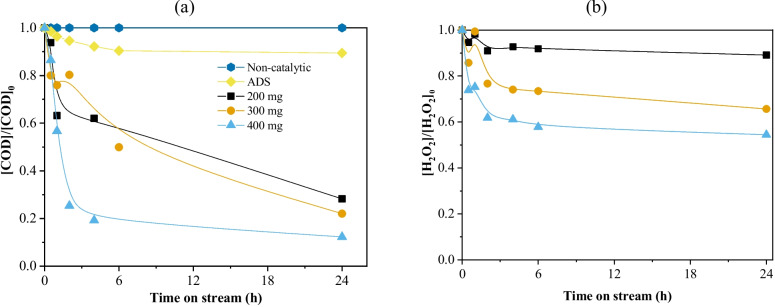
(**a**) COD evolution and (**b**) H_2_O_2_ normalized concentration considering different catalyst loads on the packed bed. Conditions: *T* = 30 °C, pH_0_ = 3.0, COD_0_ = 332 mg L^−1^, [H_2_O_2_]_0_ = 706 mg L^−1^, *m*_cat_ = 17.7–35.4 mg cm^−3^, *Q* = 1 mL min^−1^

A closer look reveals that the removal of COD with a 35.4 mg cm^−3^ catalyst load is not only higher but it also achieves a steady state much faster (Fig. 4a). From 4 h of continuous operation and onwards, a catalyst load of 35.4 mg cm^−3^ allowed achieving > 80% COD abatement, such activity being maintained up to 24 h. On the other hand, neither 17.7 mg cm^−3^ nor 26.5 mg cm^−3^ catalyst loads allowed to achieve a steady state in the 24-h operation. This result is very much related to the decomposition of hydrogen peroxide (Fig. 4b), where a 35.4 mg cm^−3^ catalyst load allowed maintaining a steady H_2_O_2_ consumption of approximately 40% from 2 h of continuous operation onwards, and under the same timeframe, a 26.5 mg cm^−3^ and 17.7 mg cm^−3^ catalyst load had only allowed a 25% and 10% H_2_O_2_ consumption, respectively.

The UV spectra in the 200–450-nm range for the raw and treated wastewater was also recorded (Figure S9). As expected, based on the results discussed above, the absorbance of the wastewater treated using 35.4 mg cm^−3^ catalyst load is considerably lower than the raw wastewater and lower than non-catalytic run (N.C.), adsorption run, and runs carried out with other catalyst loads.

The comparison between the results obtained under continuous and batch operation is not the main goal of the present work. Batch operation was indeed used as a tool to select the best-performing catalyst. In fact, given the different conditions used (i.e., IBU in distilled water in batch mode versus real wastewater in continuous mode), the direct comparison is rather complicated. Nevertheless, the operation under continuous flow with a catalyst load of 35.4 mg cm^−3^ allowed a removal rate of COD in an average of 16.8 mg_O2_ h^−1^ (from 4 to 24 h of operation, when a steady state had been reached). If we calculate the theoretical COD (ThOD) removed when operating at batch mode using IBU as the model pollutant, the removal reached was 1.2 mg_O2_ h^−1^ during the 6 h of operation. While batch reactors may be easier to operate (Al Azri et al. 2022), continuous reactors offer some advantages related to better mass and heat transfer, reduced operational cost, and no accumulation of intermediates or by-products that could lead to catalyst deactivation (Bukhtiyarova et al. 2023). These advantages increase the efficiency obtained under continuous processes (Al Azri et al. 2022).

#### Effect of the initial pH

**Fig. 5:**
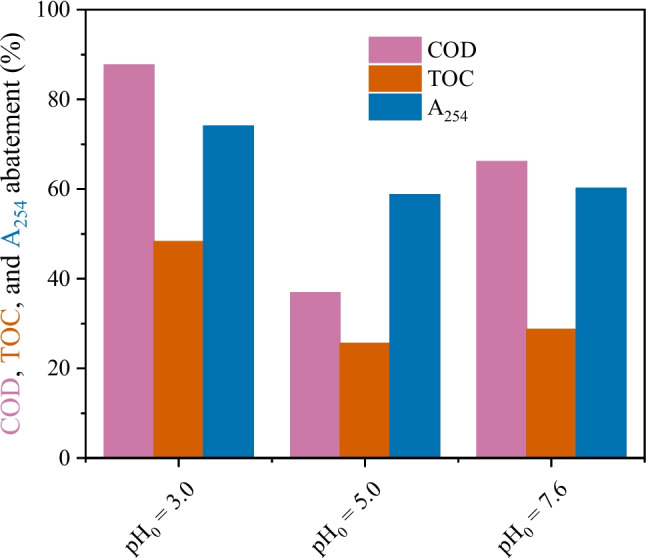
COD, TOC, and A_254_ removals considering different pH after 24 h of continuous operation. Conditions: *T* = 30 °C, COD_0_ = 332 mg L^−1^, [H_2_O_2_]_0_ = 706 mg L^−1^, *m*_cat_ = 35.4 mg cm^−3^, *Q* = 1 mL min.^−1^

The effect of the initial pH of the hospital wastewater was then studied considering the highest catalyst load in the packed bed (35.4 mg cm^−3^). The acidic pH was chosen due to the expected higher rate of hydrogen peroxide decomposition into hydroxyl radical conversion efficiency in mild acidic media (Fischbacher et al. 2017). The results are shown in Fig. 5. The pH strongly affected TOC and COD abatement, with an inverse correlation between the parameters. Higher TOC and COD abatements (48% and 88%, respectively) are achieved with the lowest initial pH, whereas the worst performance is obtained with the highest pH. Previous literature already reported that H_2_O_2_ conversion is improved in mild acidic conditions (Fischbacher et al. 2017), which is consistent with the results found in the present study. There is a strong inverse correlation between pH and COD removal (*r*^2^ = 0.99, Figure S10a), but for TOC and *A*_254_ removals, the correlation was not so evident (*r*^2^ = 0.79 and 0.76, respectively, Figure S10b,c). Higher COD removal at acidic pH has been previously reported for simulated continuous reactors (Lu et al. 2015) as well as for batch CWPO for real wastewater (Freitas et al. 2022).

**Fig. 6:**
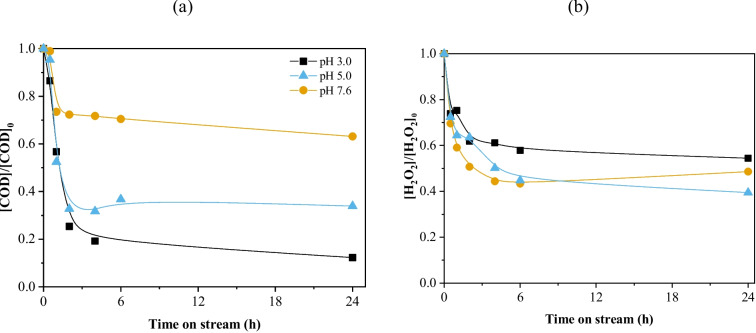
(**a**) COD evolution and (**b**) H_2_O_2_ normalized concentration considering different initial pH. Conditions: *T* = 30 °C, COD_0_ = 332 mg L^−1^, [H_2_O_2_]_0_ = 706 mg L^−1^, m_cat_ = 35.4 mg cm^−3^; *Q* = 1 mL min.^−1^

The COD profile evidences the effect of the initial wastewater pH on the COD removal (Fig. 6a). At the natural pH of the wastewater (7.6), the COD removal is very low, stabilizing at about 25% removal after 2 h of continuous operation. An initial pH of 5 improves performance, stabilizing at around 65% COD removal after 2 h of continuous operation. An initial pH of 3 results in around 80% COD removal after 4 h of continuous operation. Similar behavior has been previously reported for real wastewater treatments regarding COD removal profile and pH (Freitas et al. 2022). An inverse trend was observed for the H_2_O_2_ decomposition (Fig. 6b), where higher pH (7.6 and 5) resulted in slightly higher consumption of the oxidant source compared to an initial pH of 3. In fact, pure decomposition of H_2_O_2_ is not an accurate measure of the efficiency of the catalysts, as the H_2_O_2_ can be decomposed into other non-desirable species (Yu et al. 2020). The pH greatly affects the interaction between hydroxyl radicals and the naturally occurring radical scavengers. It has been reported that pH above 6.35 would favor the scavenging effect of hydroxyl radicals by carbonate ions (Ribeiro et al. 2017a). As the effluent under study contains carbonate ions, the lower COD removal observed for a pH of 7.6 can be attributed to their presence. Another important consideration is that the selective decomposition of H_2_O_2_ into the hydroxyl radicals is maximized in the pH range of 2–4 (Fischbacher et al. 2017). Therefore, the lower COD removal obtained for a working pH of 5 can be attributed to the formation of non-oxidizing species, such as water and oxygen.

The UV spectra of the wastewater after treatment are displayed in Figure S11. An initial pH of 3 resulted in a lower absorbance compared to pH of 7.6 or 5. Color reduction was also achieved as seen in the inset figure.

#### Effect of the temperature

**Fig. 7:**
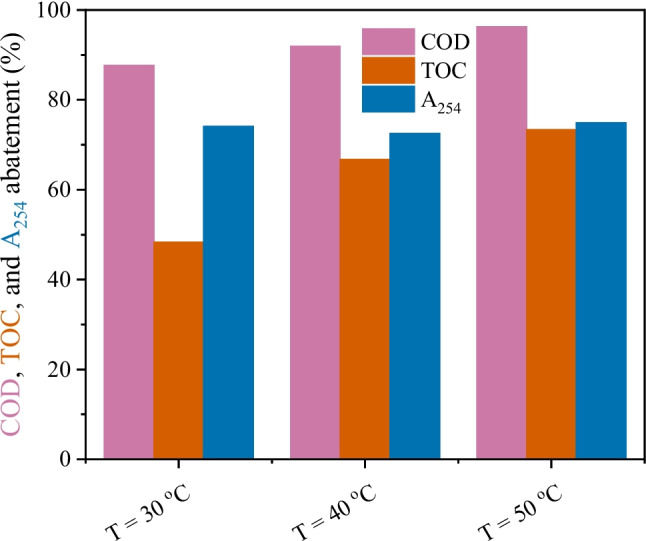
COD, TOC, and A_254_ removals considering different temperatures after 24 h of continuous operation. Conditions: pH_0_ = 3.0, COD_0_ = 332 mg L^−1^, [H_2_O_2_]_0_ = 706 mg L^−1^, *m*_cat_ = 35.4 mg cm^−3^, *Q* = 1 mL min.^−1^

CWPO can be carried out at mild temperatures. Nevertheless, temperature also affects the performance of the CWPO treatment. Therefore, the effect of the temperature of the reaction medium was explored, and the results are depicted in Fig. 7. Despite COD removal being already significant at 30 °C (> 85%), the results revealed that COD abatement increases with temperature (*r*^2^ = 0.99, Figure S12a). TOC removal is also affected by temperature (*r*^2^ = 0.93, Figure S12b), with a temperature increase from 30 to 50 °C resulting in a 1.5-fold increase in TOC removal (from ca. 50% to 75%, respectively). The removal of A_254_ was not affected by the temperature, with removals in the range 72–75% being obtained regardless of the temperature. Previous reports on the increased removal of TOC and COD with increased temperature in continuous reaction systems have been reported (Lu et al. 2015; Huang et al. 2021).

**Fig. 8:**
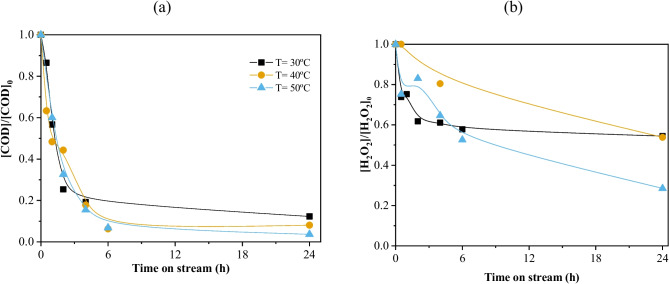
(**a**) COD evolution and (**b**) H_2_O_2_ normalized concentration considering different initial temperatures. Conditions: pH_0_ = 3.0, COD_0_ = 332 mg L^−1^, [H_2_O_2_]_0_ = 706 mg L^−1^, *m*_cat_ = 35.4 mg cm^−3^, *Q* = 1 mL min.^−1^

The COD profile (Fig. 8) is very similar in the first 4 h of continuous operation regardless of the temperature. After 4 h of continuous operation, it is possible to see the COD removal converging towards stabilization for the reactions carried out at 30 °C at around 85–87% removal. On the other hand, the reactions carried out at 40 and 50 °C only start to stabilize after 6 h of continuous operation, reaching conversions higher than 90%.

The UV spectra of the effluent are given in Figure S13. A similar absorbance at wavelengths superior to 230 nm can be observed regardless of the temperature, as expected due to similar removals of *A*_254_. However, at wavelengths lower than 230 nm, an increased absorption effluents coming from reactions at 40 and 50 °C can be seen. The absorbance at 230 nm is sensitive to the conformation of proteins that may exist in pharmaceutical wastewater. With increasing temperature, most proteins undergo denaturation when heated, explaining the different absorption spectra seen (Liu et al. 2009).

#### Stability of CoFe/CX

**Fig. 9:**
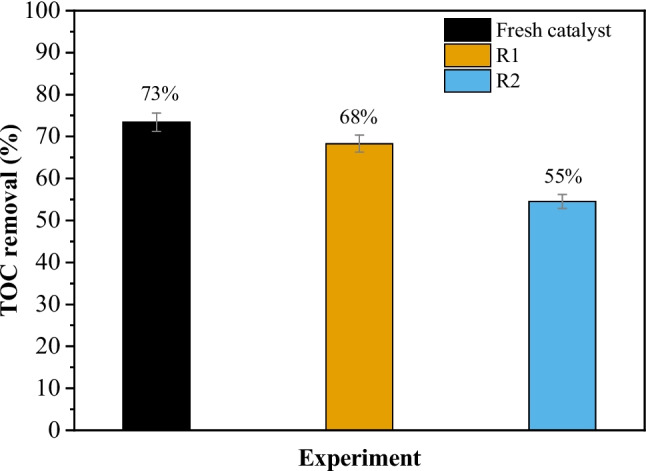
TOC removal after reutilization runs. Conditions: pH_0_ = 3.0, COD_0_ = 332 mg L^−1^, [H_2_O_2_]_0_ = 706 mg L^−1^, *m*_cat_ = 35.4 mg cm^−3^, *T* = 50 °C, *Q* = 1 mL min.^−1^

The CoFe/CX sample was used in subsequent runs to evaluate its stability. After the initial reaction was terminated, the catalyst was slightly washed with distilled water and dried in the oven at 110 °C and reused in the following reaction. The results are shown in Fig. 9. As observed, there was a slight reduction of TOC removal from the fresh catalyst to the first reuse (from 73 to 68%) and a more significant reduction in the second reutilization run (from 68 to 55%). The absorption of the wastewater (Figure S14) also changed for different runs, with the run using fresh catalyst resulting in the lowest absorption and the second reutilization run resulting in the highest absorption (but still lower than the raw wastewater). The loss of activity may be ascribed to a partial oxidation of the carbon surface (Diaz De Tuesta et al. 2021), poisoning of the catalyst surface (Diaz de Tuesta et al. 2023) by organic matter or other components from the wastewater, or leaching of active species (Lopez-Arago et al. 2024). More studies are required to understand the reason behind the loss of activity upon each cycle.

## Conclusions

The screening results demonstrated that the bimetallic material significantly outperformed single-phase catalysts. The stability tests confirmed that the best catalyst, CoFe/CX, maintained its performance over time. This catalyst was subsequently used to treat actual hospital wastewater under continuous operation. The optimal conditions for this process were determined to be an initial pH of 3.0, a catalyst load of 35.4 mg cm^−3^ in the packed bed, and a temperature of 50 °C. Under these conditions, maximum removals of total organic carbon (TOC) and chemical oxygen demand (COD) were 73% and 96%, respectively. Overall, the results suggest that continuous CWPO has significant potential for application in treating hospital wastewater, providing an effective alternative to conventional methods.

## Supplementary information

Below is the link to the electronic supplementary material.

Supplementary file1 (DOCX 2434 KB)

## Data Availability

The authors declare that the data supporting the findings of this study are available within the paper and its supplementary information files. Should any raw data files be needed in another format they are available from the corresponding author upon reasonable request.

## References

[CR1] Al Azri N, Patel R, Ozbuyukkaya G, et al (2022) Batch-to-Continuous transition in the specialty chemicals industry: impact of operational differences on the production of dispersants. Chemical Engineering Journal 445:. 10.1016/j.cej.2022.136775

[CR2] del Álamo AC, González C, Pariente MI, et al (2020) Fenton-like catalyst based on a reticulated porous perovskite material: activity and stability for the on-site removal of pharmaceutical micropollutans in a hospital wastewater. Chemical Engineering Journal 401:. 10.1016/j.cej.2020.126113

[CR3] Atugoda T, Vithanage M, Wijesekara H, et al (2021) Interactions between microplastics, pharmaceuticals and personal care products: Implications for vector transport. Environ Int 149 10.1016/j.envint.2020.106367 33497857

[CR4] Bautista P, Mohedano AF, Casas JA et al (2011) Highly stable Fe/γ-Al2O3 catalyst for catalytic wet peroxide oxidation. J Chem Technol Biotechnol 86:497–504. 10.1002/jctb.2538

[CR5] Bukhtiyarova M V., Nuzhdin AL, Bukhtiyarova GA (2023) Comparative study of batch and continuous flow reactors in selective hydrogenation of functional groups in organic compounds: what is more effective? Int J Mol Sci 24 10.3390/ijms241814136 PMC10531935 37762440

[CR6] Chakraborty P, Shappell NW, Mukhopadhyay M et al (2021) Surveillance of plasticizers, bisphenol A, steroids and caffeine in surface water of River Ganga and Sundarban wetland along the Bay of Bengal: occurrence, sources, estrogenicity screening and ecotoxicological risk assessment. Water Res 190:116668. 10.1016/j.watres.2020.116668 33285458 10.1016/j.watres.2020.116668

[CR7] Freitas BG, Roman FF, de Tuesta JLD et al (2022) Assessment of pretreatments for highly concentrated leachate waters to enhance the performance of catalytic wet peroxide oxidation with sustainable low-cost catalysts. Catalysts 12:238. 10.3390/catal12020238

[CR8] Diaz de Tuesta JL, Quintanilla A, Casas JA et al (2020) The pH effect on the kinetics of 4-nitrophenol removal by CWPO with doped carbon black catalysts. Catal Today 356:216–225. 10.1016/j.cattod.2019.08.033

[CR9] Diaz de Tuesta JL, Silva AS, Roman FF et al (2023) Polyolefin-derived carbon nanotubes as magnetic catalysts for wet peroxide oxidation of paracetamol in aqueous solutions. Catal Today 419:114162. 10.1016/j.cattod.2023.114162

[CR10] Diaz De Tuesta JL, Saviotti MC, Roman FF et al (2021) Assisted hydrothermal carbonization of agroindustrial byproducts as effective step in the production of activated carbon catalysts for wet peroxide oxidation of micro-pollutants. J Environ Chem Eng 9:105004. 10.1016/j.jece.2020.105004

[CR11] Felis E, Kalka J, Sochacki A, et al (2020) Antimicrobial pharmaceuticals in the aquatic environment - occurrence and environmental implications. Eur J Pharmacol 866:. 10.1016/j.ejphar.2019.172813 10.1016/j.ejphar.2019.172813 31751574

[CR12] Fischbacher A, von Sonntag C, Schmidt TC (2017) Hydroxyl radical yields in the Fenton process under various pH, ligand concentrations and hydrogen peroxide/Fe(II) ratios. Chemosphere 182:738–744. 10.1016/j.chemosphere.2017.05.039 28531840 10.1016/j.chemosphere.2017.05.039

[CR13] Gallego-Ríos SE, Peñuela GA (2021) Evaluation of ibuprofen and diclofenac in the main rivers of Colombia and striped catfish Pseudoplatystoma magdaleniatum. Environ Monit Assess 193:. 10.1007/s10661-021-08922-5 10.1007/s10661-021-08922-5 33755811

[CR14] Gong H, Chu W, Huang Y, et al (2021) Solar photocatalytic degradation of ibuprofen with a magnetic catalyst: effects of parameters, efficiency in effluent, mechanism and toxicity evolution. Environmental Pollution 276:. 10.1016/j.envpol.2021.116691 10.1016/j.envpol.2021.116691 33601200

[CR15] Guari NMC, Silva AS, Diaz de Tuesta JL, et al (2022) Magnetic CoFe2O4@carbon yolk-shell nanoparticles as catalysts for the catalytic wet peroxide oxidation of paracetamol: kinetic insights. 10.30955/gnj.004309

[CR16] Huaccallo-Aguilar Y, Álvarez-Torrellas S, Gil M V., et al (2021a) Insights of emerging contaminants removal in real water matrices by CWPO using a magnetic catalyst. J Environ Chem Eng 9:. 10.1016/j.jece.2021.106321

[CR17] Huaccallo-Aguilar Y, Álvarez-Torrellas S, Larriba M, et al (2021b) Naproxen removal by CWPO with Fe3O4/multi-walled carbon nanotubes in a fixed-bed reactor. J Environ Chem Eng 9:. 10.1016/j.jece.2021.105110

[CR18] Huaccallo-Aguilar Y, Diaz de Tuesta JL, Álvarez-Torrellas S, et al (2021c) New insights on the removal of diclofenac and ibuprofen by CWPO using a magnetite-based catalyst in an up-flow fixed-bed reactor. J Environ Manage 281:. 10.1016/j.jenvman.2020.111913 10.1016/j.jenvman.2020.111913 33418391

[CR19] Huang H, Zhang H, Yan Y (2021) Preparation of novel catalyst-free Fe3C nanocrystals encapsulated NCNT structured catalyst for continuous catalytic wet peroxide oxidation of phenol. J Hazard Mater 407:. 10.1016/j.jhazmat.2020.124371 10.1016/j.jhazmat.2020.124371 33248822

[CR20] Inchaurrondo N, Cechini J, Font J, Haure P (2012) Strategies for enhanced CWPO of phenol solutions. Appl Catal B 111–112:641–648. 10.1016/j.apcatb.2011.11.019

[CR21] Li J, You J, Wang Z, et al (2022) Application of α-Fe2O3-based heterogeneous photo-Fenton catalyst in wastewater treatment: a review of recent advances. J Environ Chem Eng 10

[CR22] Liu P-F, Avramova LV, Park C (2009) Revisiting absorbance at 230nm as a protein unfolding probe. Anal Biochem 389:165–170. 10.1016/j.ab.2009.03.028 19318083 10.1016/j.ab.2009.03.028

[CR23] Liu Z, Shen Q, Zhou C, et al (2018) Kinetic and mechanistic study on catalytic decomposition of hydrogen peroxide on carbon-nanodots/graphitic carbon nitride composite. Catalysts 8:. 10.3390/catal8100445

[CR24] Liu N, Jin X, Feng C, et al (2020) Ecological risk assessment of fifty pharmaceuticals and personal care products (PPCPs) in Chinese surface waters: a proposed multiple-level system. Environ Int 136:. 10.1016/j.envint.2019.105454 10.1016/j.envint.2019.105454 32032889

[CR25] Lofrano G, Faiella M, Carotenuto M, et al (2021) Thirty contaminants of emerging concern identified in secondary treated hospital wastewater and their removal by solar Fenton (like) and sulphate radicals-based advanced oxidation processes. J Environ Chem Eng 9:. 10.1016/j.jece.2021.106614

[CR26] Lopez-Arago N, Munoz M, de Pedro ZM, Casas JA (2024) Natural magnetite as an effective and long-lasting catalyst for CWPO of azole pesticides in a continuous up-flow fixed-bed reactor. Environ Sci Pollut Res 31:29148–29161. 10.1007/s11356-024-33065-8 10.1007/s11356-024-33065-8 PMC11058975 38568307

[CR27] Lu M, Yao Y, Gao L, et al (2015) Continuous treatment of phenol over an Fe2O3/γ-Al2O3 catalyst in a fixed-bed reactor. Water Air Soil Pollut 226:. 10.1007/s11270-015-2363-0

[CR28] Márquez JJR, Levchuk I, Sillanpää M (2018) Application of catalytic wet peroxide oxidation for industrial and urban wastewater treatment: a review. Catalysts 8

[CR29] Mello F V., Cunha SC, Fogaça FHS, et al (2022) Occurrence of pharmaceuticals in seafood from two Brazilian coastal areas: Implication for human risk assessment. Science of the Total Environment 803:. 10.1016/j.scitotenv.2021.149744 10.1016/j.scitotenv.2021.149744 34482147

[CR30] Moreno-Castilla C, Maldonado-Hódar FJ (2005) Carbon aerogels for catalysis applications: an overview. Carbon N Y 43:455–465

[CR31] Morin-Crini N, Lichtfouse E, Liu G et al (2022) Worldwide cases of water pollution by emerging contaminants: a review. Environ Chem Lett 20:2311–2338

[CR32] Munoz M, Mora FJ, de Pedro ZM et al (2017) Application of CWPO to the treatment of pharmaceutical emerging pollutants in different water matrices with a ferromagnetic catalyst. J Hazard Mater 331:45–54. 10.1016/j.jhazmat.2017.02.017 28242528 10.1016/j.jhazmat.2017.02.017

[CR33] Nawaz M, Khan AA, Hussain A, et al (2020) Reduced graphene oxide−TiO2/sodium alginate 3-dimensional structure aerogel for enhanced photocatalytic degradation of ibuprofen and sulfamethoxazole. Chemosphere 261:. 10.1016/j.chemosphere.2020.127702 10.1016/j.chemosphere.2020.127702 32750619

[CR34] Noguera-Oviedo K, Aga DS (2016) Lessons learned from more than two decades of research on emerging contaminants in the environment. J Hazard Mater 316:242–251 27241399 10.1016/j.jhazmat.2016.04.058

[CR35] Paparo R, Di Serio M, Roviello G, et al (2024) Geopolymer-based materials for the removal of ibuprofen: a preliminary study. Molecules 29:. 10.3390/molecules29102210 10.3390/molecules29102210 PMC11124334 38792071

[CR36] Pot EJ, Milakovic M, Chaumot A et al (2022). Pharmaceutical Pollution of the World’s Rivers. 10.1073/pnas.2113947119/-/DCSupplemental

[CR37] Rathi BS, Kumar PS, Vo DVN (2021) Critical review on hazardous pollutants in water environment: occurrence, monitoring, fate, removal technologies and risk assessment. Science of the Total Environment 797:. 10.1016/j.scitotenv.2021.149134 10.1016/j.scitotenv.2021.149134 34346357

[CR38] Rayaroth MP, Boczkaj G, Aubry O, et al (2023) Advanced oxidation processes for degradation of water pollutants—ambivalent impact of carbonate species: a review. Water (Switzerland) 15

[CR39] Ribeiro RS, Silva AMT, Pastrana-Martínez LM et al (2015) Graphene-based materials for the catalytic wet peroxide oxidation of highly concentrated 4-nitrophenol solutions. Catal Today 249:204–212. 10.1016/j.cattod.2014.10.004

[CR40] Ribeiro RS, Frontistis Z, Mantzavinos D et al (2016a) Magnetic carbon xerogels for the catalytic wet peroxide oxidation of sulfamethoxazole in environmentally relevant water matrices. Appl Catal B 199:170–186. 10.1016/j.apcatb.2016.06.021

[CR41] Ribeiro RS, Silva AMT, Figueiredo JL et al (2016b) Catalytic wet peroxide oxidation: a route towards the application of hybrid magnetic carbon nanocomposites for the degradation of organic pollutants. A Review Appl Catal B 187:428–460. 10.1016/j.apcatb.2016.01.033

[CR42] Ribeiro RS, Rodrigues RO, Silva AMT et al (2017a) Hybrid magnetic graphitic nanocomposites towards catalytic wet peroxide oxidation of the liquid effluent from a mechanical biological treatment plant for municipal solid waste. Appl Catal B 219:645–657. 10.1016/j.apcatb.2017.08.013

[CR43] Ribeiro RS, Silva AMT, Figueiredo JL et al (2017b) The role of cobalt in bimetallic iron-cobalt magnetic carbon xerogels developed for catalytic wet peroxide oxidation. Catal Today 296:66–75. 10.1016/j.cattod.2017.06.023

[CR44] Santos Silva A, Seitovna Kalmakhanova M, Kabykenovna Massalimova B et al (2019) Wet peroxide oxidation of paracetamol using acid activated and Fe/Co-pillared clay catalysts prepared from natural clays. Catalysts 9:705. 10.3390/catal9090705

[CR45] Santos Silva A, Roman FF, da Silva APF, et al (2024) Reactive materials and solutions towards treatment and reuse of waters with contaminants of emerging concern. In: Galvão João Rafael da Costa Sanches and Brito P and NF dos S and AH de A and MS de JM and NC (ed) Proceedings of the 3rd International Conference on Water Energy Food and Sustainability (ICoWEFS 2023). Springer Nature Switzerland, Cham, pp 477–488

[CR46] Shah AI, Din Dar MU, Bhat RA, et al (2020) Prospectives and challenges of wastewater treatment technologies to combat contaminants of emerging concerns. Ecol Eng 152

[CR47] Silva AS, Roman FF, Dias AV et al (2023) Hybrid multi-core shell magnetic nanoparticles for wet peroxide oxidation of paracetamol: application in synthetic and real matrices. J Environ Chem Eng 11:110806. 10.1016/j.jece.2023.110806

[CR48] Silva AS, Diaz de Tuesta JL, Henrique A, et al (2024) 3D printed photopolymer derived carbon catalysts for enhanced wet peroxide oxidation. Chemical Engineering Journal 156574. 10.1016/j.cej.2024.156574

[CR49] Sousa JCG, Ribeiro AR, Barbosa MO et al (2018) A review on environmental monitoring of water organic pollutants identified by EU guidelines. J Hazard Mater 344:146–162 29032095 10.1016/j.jhazmat.2017.09.058

[CR50] Starling MCVM, Amorim CC, Leão MMD (2019) Occurrence, control and fate of contaminants of emerging concern in environmental compartments in Brazil. J Hazard Mater 17–36. 10.1016/j.jhazmat.2018.04.043 10.1016/j.jhazmat.2018.04.043 29728279

[CR51] Sun Y, Yang Z, Tian P et al (2019) Oxidative degradation of nitrobenzene by a Fenton-like reaction with Fe-Cu bimetallic catalysts. Appl Catal B 244:1–10. 10.1016/j.apcatb.2018.11.009

[CR52] Verlicchi P (2021) Trends, new insights and perspectives in the treatment of hospital effluents. Curr Opin Environ Sci Health 19 10.1016/j.coesh.2020.10.005 PMC7571420 33103011

[CR53] Vieira Y, Pereira HA, Leichtweis J, et al (2021) Effective treatment of hospital wastewater with high-concentration diclofenac and ibuprofen using a promising technology based on degradation reaction catalyzed by Fe0 under microwave irradiation. Science of the Total Environment 783:. 10.1016/j.scitotenv.2021.146991 10.1016/j.scitotenv.2021.146991 33865131

[CR54] Yu Y, Tang Z, Wang J et al (2020) Insights into the efficiency of hydrogen peroxide utilization over titanosilicate/H2O2 systems. J Catal 381:96–107. 10.1016/j.jcat.2019.09.045

[CR55] Zapata NI, Peñuela GA (1951) Modified QuEChERS/UPLC-MS/MS method to monitor triclosan, ibuprofen, and diclofenac in fish Pseudoplatystoma magdaleniatum. 10.1007/s12161-020-01951-9/Published

[CR56] Zazo JA, Casas JA, Mohedano AF, Rodríguez JJ (2006) Catalytic wet peroxide oxidation of phenol with a Fe/active carbon catalyst. Appl Catal B 65:261–268. 10.1016/j.apcatb.2006.02.008

[CR57] Zhang N, Liu X, Pan L et al (2021) Evaluation of ibuprofen contamination in local urban rivers and its effects on immune parameters of juvenile grass carp. Fish Physiol Biochem 47:1405–1413. 10.1007/s10695-021-00987-w 34291405 10.1007/s10695-021-00987-w

[CR58] Zhou Z, Wu Y, Kuang Y et al (2024) Assessment of ibuprofen toxicity and removal potential of Chlorella vulgaris. Bioremediat J 28:213–221. 10.1080/10889868.2022.2138823

