# Supplementary material for: Beyond batch experiments: unveiling the potential of bimetallic carbon xerogels for catalytic wet peroxide oxidation of hospital wastewater in continuous mode
Source: Environ Sci Pollut Res Int. 2024 Nov 22;31(57):65208–19. doi: 10.1007/s11356-024-35546-2 (PMC11631992; doi:10.1007/s11356-024-35546-2)
Supplement: Supplementary file 1 — Supplementary file1 (DOCX 2434 KB) [file 11356_2024_35546_MOESM1_ESM.docx]

**SUPPLEMENTARY INFORMATION FOR:**

**Beyond batch experiments: Unveiling the potential of bimetallic carbon xerogels for catalytic wet peroxide oxidation of hospital wastewater in continuous mode**

Adriano dos Santos Silva^1,2,3,4,^, Fernanda Fontana Roman^1,2,3,4^, Rui Sérgio Ribeiro^3,4^, Juan Garcia^5*^, Helder Teixeira Gomes^1,2,*^

^1^ Centro de Investigação de Montanha (CIMO), Instituto Politécnico de Bragança, Campus de Santa Apolónia, 5300‑253 Bragança, Portugal;

^2^ Laboratório Associado para a Sustentabilidade e Tecnologia em Regiões de Montanha (SusTEC), Instituto Politécnico de Bragança, Campus de Santa Apolónia, 5300 253 Bragança, Portugal;

^3^ LSRE-LCM – Laboratory of Separation and Reaction Engineering - Laboratory of Catalysis and Materials, Faculty of Engineering, University of Porto, Rua Dr. Roberto Frias, 4200-465 Porto, Portugal;

^4^ ALiCE – Associate Laboratory in Chemical Engineering, Faculty of Engineering, University of Porto, Rua Dr. Roberto Frias, 4200-465 Porto, Portugal;

^5^ Catalysis and Separation Procecesses Group (CyPS), Chemical Engineering and Materials Department, Complutense University of Madrid, Avda. Complutense s/n, Madrid, Spain

^*^ Corresponding authors:

Tel.: +351 273 303 110; Fax: +351 273 313 051;

E-mail addresses: jgarciar@ucm.es, htgomes@ipb.pt


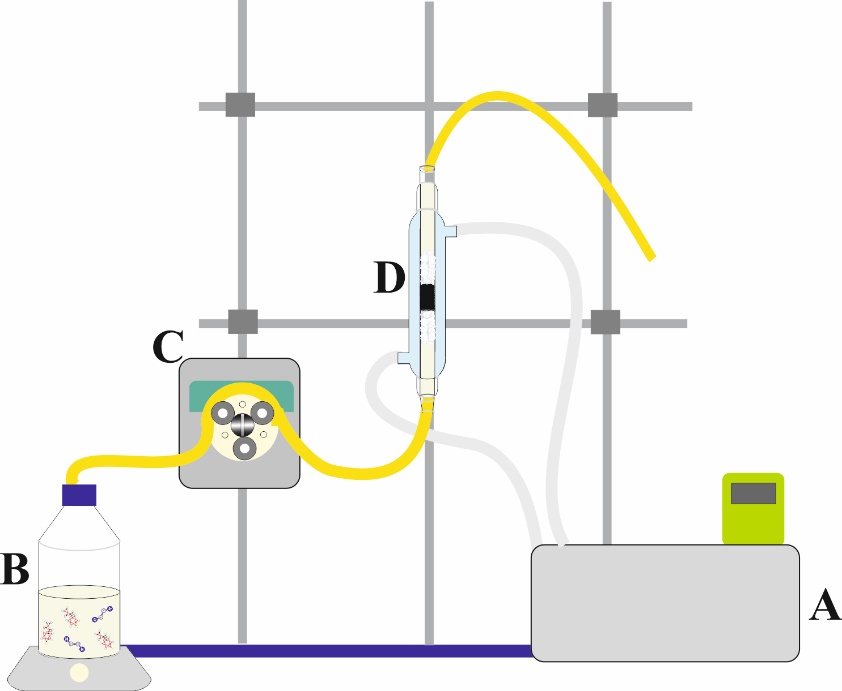


Figure S1. Continuous system used for the CWPO experiments. (A) is the recirculation bath used to heat the reactor, (B) is the model hospital wastewater and oxidant solution, (C) is the peristaltic pump, and (D) is the reactor loaded with catalyst.

| (a) | (b) |
| --- | --- |
|  |  |

Figure S2. (a) COD calibration curve and (b) interference of H_2_O_2_ in the COD determination.

**Text S1- Carbon xerogels synthesis and characterization procedures**

CX was prepared following a procedure described our previous works [1]. The polycondensation of resorcinol with formaldehyde was explored to achieve the desired material. In brief, 13.5 mL of formaldehyde solution was added in a flask previously loaded with 9.91 g of resorcinol. Next, the mixture pH was adjusted to 6.1 by means of NaOH solutions (1 mol L^-1^ and 0.02 mol L^-1^). The final mixture went through the gelation step for 3 days at 85 °C without the addition of catalyst, and the gel was recovered and grinded to powder. The recovered material was separated and the fraction with particle sizes in the range 0.106 – 0.25 mm was separated, dried in oven according to the thermal treatment illustrated in Figure S3, and annealed under N_2_ flow (100 cm^3^ min^-1^) according to the program illustrated in Figure S4, resulting in a material named CX.

The carbon xerogels prepared with embedded metals followed the same methodology described previously, except for adding metal precursor in the resorcinol solution. For the synthesis of CX/Fe, iron (III) chloride was added to the resorcinol solution to reach a molar ratio Fe/resorcinol = 0.05, and the solution was stirred in an orbital shaker for 2 h before the addition of the formaldehyde solution. For the synthesis of CX/CoFe, iron(III) chloride (Fe/resorcinol = 0.05) and cobalt (II) chloride (Fe/Co = 2) were added to the resorcinol solution, following the same steps previously mentioned. The molar ratio Fe/Co was chosen according to other works dealing with synthesizing iron-copper bimetallic nanoparticles embedded within mesoporous carbon materials [2].

The textural characteristics of the carbon xerogel materials were assessed using N_2_ adsorption-desorption isotherms at -196°C with a Quantachrome NOVA 4200e adsorption analyzer. Prior to analysis, samples were outgassed for 6 hours at 130°C. The specific surface area (S_BET_) was calculated using the Brunauer-Emmett-Teller (BET) equation. Micropore volume (V_micro_) and non-microporous surface area (S_meso_) were determined via the t-method with a suitable standard isotherm. Total pore volume (V_total_) was obtained from the N_2_ adsorption at a relative pressure of p/p^0^ = 0.995. The average pore diameter (d_pore_) was estimated using Equation S1, assuming cylindrical pores with most surface area from pore walls:

$$d_{pore}=4\cdot\frac{V_{total}}{S_{BET}} (S1)$$

The point of zero charge (pH_PZC_) was determined by varying initial pH (2-11) of solutions using 1.0 mol/L HCl or NaOH and 20 mL of 0.01 mol/L NaCl as the electrolyte. The final pH was measured after 48 hours of stirring at room temperature, with pH_PZC_ determined at the intersection of the final vs. initial pH curve with the line final pH = initial pH. X-ray photoelectron spectroscopy (XPS) was conducted using a Kratos Axis Ultra HAS with a monochromatic Al X-ray source (1486.7 eV) at 15 kV (90W) in lens hybrid mode. Charge correction was based on the C 1s peak (285 eV), and after Shirley background subtraction, peaks were fitted with Gaussian curves using CasaXPS software.

Scanning electron microscopy (SEM) images were captured in secondary electron (SE) and backscattered electron (BSE) modes using a FEI Quanta 400FEG ESEM/EDAX Genesis X4M instrument equipped with an Energy Dispersive Spectrometer (EDS). ImageJ software was employed to analyze at least 110 counts to estimate the size of the primary carbon microspheres in the carbon xerogel materials, and a minimum of 65 counts were analyzed to determine the size of the metal particles embedded within the structure.

Figure S3. Ramp used for drying the materials

Figure S4. Ramp used for annealing the materials.


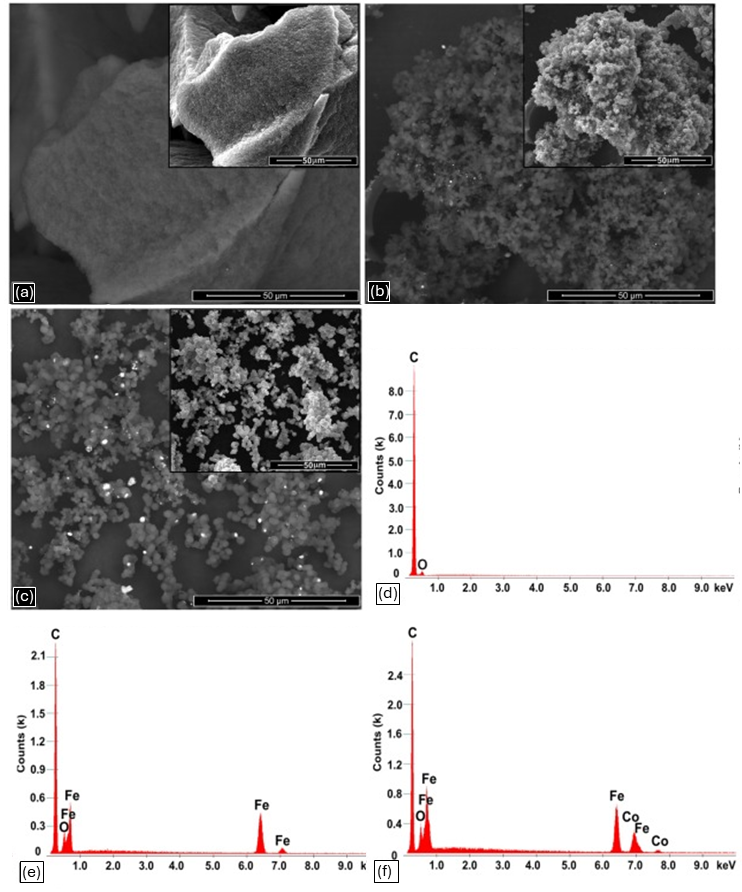


Figure S5. SEM micrographs of (a) CX, (b) CX/Fe, (c) CX/CoFe, obtained in (main) BSE and (inset) SE mode. EDX spectra of (d) CX, (e) CX/Fe, and (f) CX/CoFe. Adapted from Applied B Environmental, 199, 170-186 (2016) with permission from Elsevier (license number 5847641479567).


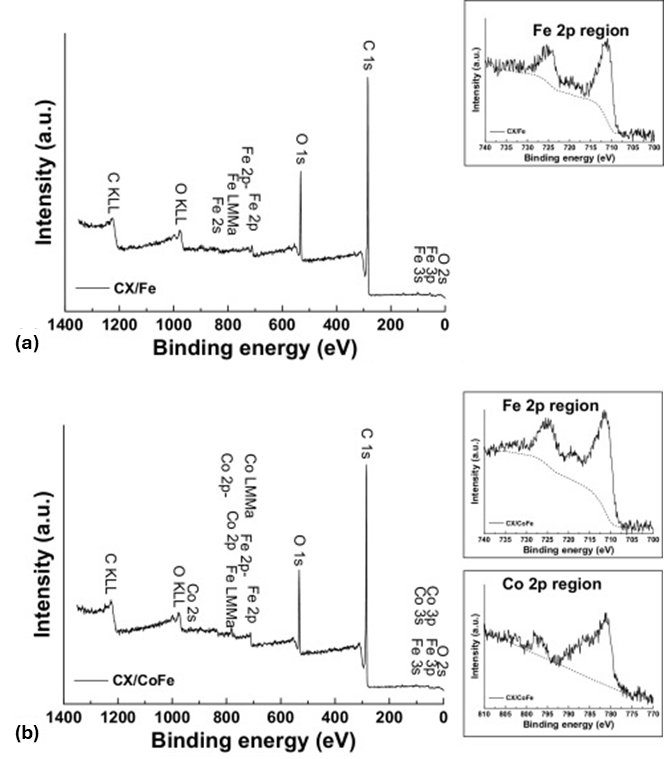


Figure S6.  XPS spectra of (a) CX/Fe and (c) CX/CoFe. Insets: detailed XPS spectra of Fe 2p and/or Co 2p regions. Reproduced from Applied B Environmental, 193, 170-186 with permission from Elsevier (license number 5847641479567).

Figure S7. Correlation between adsorption capacity and (a) surface area and (b) pH_PZC_.

Figure S8. Correlations between catalyst load in continuous system and (a) COD, (b) TOC and (c) A_254_ removals.

Figure S9. UV-spectra of the wastewater before and after CWPO treatment considering different catalyst loads.

Figure S10. Correlations between pH in continuous system and (a) COD, (b) TOC and (c) A_254_ removals.

Figure S11. UV-spectra of the wastewater before and after CWPO treatment considering different initial pHs and inset with the difference in color of the effluent before and after treatment.

Figure S12. Correlations between temperature in continuous system and (a) COD and (b) TOC removals.

Figure S13 UV-spectra of the wastewater before and after CWPO treatment considering different temperatures.

Figure S14 UV-spectra of the wastewater before and after CWPO treatment for catalyst reutilization.

Table S1. Operating conditions in the continious CWPO process.

| **Exp.** | **Catalyst load**  **(mg)** | **H_2_O_2_** | **Temperature (°C)** | **pH_0_** |
| --- | --- | --- | --- | --- |
| **1** | ----- | Yes | 30 | 3.0 |
| **2** | 200 | ------ | 30 | 3.0 |
| **3** | 200 | Yes | 30 | 3.0 |
| **4** | 300 | Yes | 30 | 3.0 |
| **5** | 400 | Yes | 30 | 3.0 |
| **6** | 400 | Yes | 30 | 7.6 |
| **7** | 400 | Yes | 30 | 5.0 |
| **8** | 400 | Yes | 40 | 3.0 |
| **9** | 400 | Yes | 50 | 3.0 |
| **10R1** | 400 | Yes | 50 | 3.0 |
| **11R2** | 400 | Yes | 50 | 3.0 |

**Table S2.** Textural properties, pH_PZC_ and Fe and Co content of the materials.

| **Material** | **Parameter** | | | | | | | | | |
| --- | --- | --- | --- | --- | --- | --- | --- | --- | --- | --- |
|  | **S_BET_ (m^2^ g^-1^)** | **S_meso_ (m^2^ g^-1^)** | **V_mic_ (cm^3^ g^-1^)** | **Vt_otal_ (cm^3^ g^-1^)** | **V_mic_/ V_total_** | **d_pore_ (nm)** | **pH_PZC_** | **Fe (wt.%)** | **Co (wt.%)** |  |
| **CX** | 650 | 240 | 0.19 | 1.09 | 0.17 | 6.7 | 9.2 | – | – |  |
| **Fe/CX** | 510 | 90 | 0.17 | 0.46 | 0.37 | 3.6 | 6.6 | 6.5 | – |  |
| **CoFe/CX** | 530 | 40 | 0.2 | 0.3 | 0.67 | 2.3 | 7.7 | 4.6 | 2.1 |  |

**Table S3.** Relative atomic surface content of C, O, Fe and Co in the surface of the materials.

| Material | Atomic surface concentration (%) | | | | Weight surface concentration (%) | | | |
| --- | --- | --- | --- | --- | --- | --- | --- | --- |
|  | C 1s | O 1s | Fe 2p | Co 2p | C 1s | O 1s | Fe 2p | Co 2p |
| CX |  |  |  |  |  |  |  |  |
| Fe/CX | 84.82 | 14.48 | 0.7 | – | 79 | 17.97 | 3.03 | – |
| CoFe/CX | 84.56 | 13.64 | 1.19 | 0.61 | 76.01 | 16.33 | 4.97 | 2.69 |
